# Evaluación de costos y resultados de la Campaña de Erradicación de la Malaria en Colombia entre 1957 y 1969

**DOI:** 10.7705/biomedica.7518

**Published:** 2025-08-11

**Authors:** Julio César Padilla-Rodríguez, Mario J. Olivera

**Affiliations:** 1 Red de Gestión de Conocimiento, Investigación e Innovación en Malaria, Bogotá, D. C., Colombia Investigación e Innovación en Malaria Investigación e Innovación en Malaria Bogotá, D. C. Colombia; 2 Grupo de Parasitología, Dirección de Investigación en Salud Pública, Instituto Nacional de Salud, Bogotá, D. C., Colombia Instituto Nacional de Salud Dirección de Investigación en Salud Pública Instituto Nacional de Salud Bogotá, D. C. Colombia

**Keywords:** malaria, erradicación de la enfermedad, economía de la salud, costos, análisis de costo, Malaria, disease eradication, health economics, costs, cost analysis.

## Abstract

**Introducción.:**

Entre 1959 y 1969, Colombia implemento la Campaña de Erradicación de la Malaria, como parte de una estrategia global para eliminar la enfermedad.

**Objetivo.:**

Estimar los costos de la Campaña de Erradicación de la Malaria en Colombia, desde una perspectiva institucional.

**Materiales y métodos.:**

Se hizo una evaluación económica parcial con un análisis descriptivo de costos, mediante microdatos de informes oficiales del Servicio de Erradicación de la Malaria, la División de Campañas Directas y la Organización Panamericana de la Salud (1958-1969). Los costos se clasificaron según las fases operativas: ataque, vigilancia y mantenimiento; y se desglosaron en rubros, como personal, prestaciones, equipamiento y otros costos.

**Resultados.:**

El costo total de la Campaña de Erradicación de la Malaria fue de USD $41788.924 con el 74,1 % de aportes nacionales. La fase de ataque representó el 80,4 % del costo (USD $33’603.645), mientras que el 19,6 % restante (USD $8’185.279) correspondió a las fases preparatoria, de consolidación y mantenimiento. En la fase de ataque, el 40 % del gasto se asignó al personal encargado de las actividades de rociamiento intradomiciliario y, el 19 %, a la adquisición de insecticidas. El costo promedio por vivienda rociada fue de USD $2,70. Con la campaña se lograron prevenir 568.439 casos de malaria.

**Conclusiones.:**

El costo institucional de la Campaña de Erradicación de la Malaria en Colombia ascendió a USD $41788.924; la mayor parte de la financiación (74,1 %) fue de origen nacional. Aunque con la campaña se previnieron, en promedio, 41.965 casos de malaria por año, no se logró erradicar la enfermedad. Estos hallazgos resaltan la magnitud de la inversión requerida para programas de control vectorial a gran escala, y subrayan la importancia de implementar estrategias sostenibles de vigilancia y control.

## Abstract (en)

**Introduction.:**

Between 1959 and 1969, Colombia implemented the *Campaña de Erradicación de la Malaria* (Malaria Eradication Campaign) as part of a global initiative.

**Objective.:**

To estimate the institutional costs of the *Campaña de Erradicación de la Malaria* in Colombia.

**Materials and methods.:**

A partial economic evaluation was conducted to describe costs and outcomes based on microdata from reports by the *Servicio de Erradicación de la Malaria*, the *División de Campañas Directas*, and the Pan American Health Organization, from 1958 to 1969. Costs were classified according to the operational phases of the *Campaña de Erradicación de la Malaria*-attack, surveillance, and maintenance-and expenditures were categorized into personnel, benefits, equipment, and other associated costs.

**Results.:**

The total cost of the *Campaña de Erradicación de la Malaria* was USD $41788,924; 74.1% funded by national sources. The attack phase accounted for 80.4% of the total expenditure (USD $33’603,645), while the remaining USD $8’185,279 corresponded to the preparatory, consolidation, and maintenance phases. Forty percent of the attack phase expenditure was allocated to personnel responsible for spraying activities, while 19% percent was spent on insecticide acquisition. The average cost per household spraying was USD $2.70. The campaign prevented an estimated 568,439 malaria cases.

**Conclusions.:**

The institutional cost of the *Campaña de Erradicación de la Malaria* in Colombia amounted to USD $41788,924, with 74.1% corresponding to national resources. Although the campaign prevented an average of 41,965 malaria cases per year, it ultimately failed to achieve malaria eradication.

La malaria o paludismo es una enfermedad transmisible causada por parásitos del género *Plasmodium*, con una gran prevalencia en regiones tropicales y un impacto significativo en la salud pública y la economía global ^1^. Su carga socioeconómica se debe a la morbilidad elevada, las complicaciones clínicas, la discapacidad generada y la mortalidad asociada, lo que afecta la fuerza laboral y el desarrollo económico de los países endémicos ^2^^,^^3^.

 Históricamente, la malaria ha sido reconocida como un problema prioritario de salud pública, particularmente en contextos de expansión económica y explotación de recursos naturales en los países tropicales ^4^^,^^5^. Durante el siglo XX, su impacto económico comenzó a cuantificarse. En 1956, se estimó que los costos anuales asociados con la enfermedad en Colombia alcanzaban los USD $30 millones anuales de la época ^6^. A nivel global, en la década de 1950, la Organización Mundial de la Salud (OMS) estimó que la malaria afectaba a 250 millones de personas anualmente y que causaba la muerte de más de 2,5 millones. En las Américas, 100 millones de personas estaban en riesgo de infección en un área endémica de aproximadamente 23 millones de km² ^7^, mientras que, en Colombia, se reportaban 71.575 casos anuales en promedio ^8^. 

Tras la Segunda Guerra Mundial, el establecimiento de un nuevo orden económico global favoreció la creación de organismos internacionales, como la OMS, que impulsaron estrategias de erradicación de enfermedades, entre ellas, la malaria ^9^. Los programas de erradicación se justificaban por razones económicas y técnicas, como el impacto de la malaria en las economías nacionales, la viabilidad de la erradicación a corto plazo frente a los costos de control a largo plazo y la creciente resistencia al dicloro-difeniltricloroetano (DDT) ^10^.

En 1955, durante la Octava Asamblea Mundial de la Salud, la OMS asumió el liderazgo de la erradicación de la malaria, definida como la eliminación permanente de la transmisión y del reservorio infeccioso, sin posibilidad de restablecimiento ^8^. Para ello, se establecieron cuatro fases: preparación, ataque, consolidación y mantenimiento. A diferencia de la erradicación, la eliminación busca interrumpir la transmisión local en un área específica, aunque con posibilidad de reintroducción; por su parte, el control se enfoca en reducir la morbilidad y la mortalidad a niveles manejables mediante intervenciones continuas ^5^.

En 1956, Colombia y otros países latinoamericanos adoptaron la iniciativa de erradicación de la malaria en un acuerdo con la Organización Panamericana de la Salud (OPS) y el Fondo de las Naciones Unidas para la Infancia (UNICEF) ^5^. La fase de ataque se ejecutó entre 1958 y 1963, pero la sostenibilidad de las medidas fue un desafío que llevó al resurgimiento de la transmisión ^11^. Hasta la fecha, los estudios sobre el impacto económico de la Campaña de Erradicación de la Malaria en Colombia han sido limitados ^5^^,^^12^.

La transmisión de malaria en Colombia persiste con patrones endémicos y brotes recurrentes ^11^. Por esta razón, para la implementación de nuevas estrategias orientadas a su eliminación, resulta fundamental revisar experiencias previas desde una perspectiva económica. Este estudio propone un análisis retrospectivo de la Campaña de Erradicación de la Malaria, desarrollada entre 1957 y 1969, para evaluar la correspondencia entre la inversión institucional y los resultados alcanzados en términos de erradicación de la enfermedad, eficiencia operativa y sostenibilidad. Estos hallazgos aportarán evidencia útil para orientar los esfuerzos actuales dirigidos al control de la malaria en la región.

El objetivo de este estudio fue describir los costos y resultados del Programa de Erradicación de la Malaria en Colombia entre 1957 y 1969 desde una perspectiva institucional.

## Materiales y métodos

### Diseño del estudio

Se llevó a cabo una evaluación económica parcial retrospectiva, para analizar los costos y los resultados de la Campaña de Erradicación de la Malaria en Colombia entre 1957 y 1969. El análisis se hizo desde la perspectiva del proveedor de servicios de salud pública y se consideraron los costos asociados con las diferentes fases operativas de la campaña.

### Fases de la Campaña de Erradicación de la Malaria

El estudio se estructuró en función de las cuatro fases establecidas en la Campaña, como sigue:

1. *Fase preparatoria:* su objetivo fue establecer una línea epidemiológica de base sobre la distribución y la intensidad de la transmisión de la malaria. Para ello, se hizo una encuesta epidemiológica y entomológica que proporcionó información clave para la planificación operativa de la campaña. Esta fase tuvo una duración de un año (1957-1958).
2. *Fase de ataque:* buscaba la interrupción total de la transmisión y la eliminación del reservorio del parásito. Se implemento una estrategia de rociamiento intradomiciliario con DDT, con la que se logró la cobertura del 100 % de las viviendas en áreas endémicas mediante la aplicación de este insecticida en ciclos semestrales durante cinco años.
3. *Fase de consolidación y mantenimiento:* una vez reducida la transmisión, se implementaron acciones de vigilancia epidemiológica y control focalizadas en evitar la reintroducción del parásito. Se incluyeron la detección temprana y la eliminación de focos residuales, junto con estrategias para reducir los reservónos humanos mediante la administración de tratamiento antimalárico en los casos detectados.

### Identificación y medición de costos

Se recopilaron los datos de los informes oficiales del Servicio de Erradicación de la Malaria, la División de Campañas Directas y la OPS. De los informes del Servicio de Erradicación de la Malaria, se extrajo información detallada sobre gastos operativos, como salarios del personal (administrativo y de campo) y consumo de insecticidas y materiales. Los documentos de la División de Campañas Directas aportaron datos sobre la logística de la campaña asociados con costos de transporte, almacenamiento y distribución de insumos. Por su parte, los informes de la OPS permitieron identificar las fuentes y montos de financiación internacional, así como las directrices técnicas relacionadas con la asignación de recursos. Los costos se estimaron mediante un enfoque de microcosteo. Se desglosaron los recursos en distintas categorías: personal, apoyo logístico, insumos críticos, materiales y equipos, y fuentes de financiación, diferenciando entre aportes nacionales e internacionales.

### Análisis estadístico

Los datos se registraron y organizaron en Microsoft Excel^®^ y los análisis estadísticos se realizaron con R, versión 2.15 (R Development Core Team, R Foundation for Statistical Computing, Vienna, Austria). Para describir las variables incluidas en el estudio se emplearon frecuencias absolutas (número de casos observados y prevenidos) y relativas (porcentajes). Para las estimaciones de costos y proporciones, se calcularon intervalos de confianza del 95 % (IC_95%_) con el fin de reflejar la variabilidad estadística y la precisión de los resultados reportados.

Para estimar los casos prevenidos durante la Campaña de Erradicación de la Malaria, se utilizó como línea de base el promedio anual de los casos registrados en la década de 1950 (71.031 casos anuales), antes del inicio de la intervención. A partir de este valor, se estimó la incidencia esperada para cada año entre 1957 y 1969, asumiendo una incidencia constante en ausencia del programa. Los casos prevenidos se calcularon como la diferencia entre la incidencia esperada y la observada, multiplicada por la población anual en riesgo. Esta metodología corresponde a un enfoque de diferencia de incidencias ajustado por población.

Las medidas de tendencia central y de dispersión se calcularon para las variables continuas que presentaron una distribución normal según las pruebas de Shapiro-Wilk y Kolmogorov-Smirnov; para las variables categóricas, se utilizaron frecuencias absolutas y relativas. Los costos se expresaron en dólares estadounidenses (USD) de la época, aplicando la tasa representativa del mercado promedio anual vigente para cada año entre 1957 y 1969. La tasa representativa del mercado promedio en ese periodo fue de COP $13,34 por cada dólar, según los registros del Banco de la República, el Departamento Administrativo Nacional de Estadística (DANE), y la Comisión Económica para América Latina y el Caribe (CEPAL) ^13^^,^^14^.

### Análisis de sensibilidad

Para evaluar la incertidumbre en la estimación de costos, se llevó a cabo un análisis univariado de sensibilidad, considerando variaciones en los siguientes costos: adquisición de insecticidas (± 25 %) y medicamentos (± 20 %), salarios del personal de campo (-20 %, +10 %), alojamiento (± 20 %), transporte (± 30 %), equipos y suministros (± 30 %) y número de casos diagnosticados (± 20 %). Los resultados de este análisis se representaron mediante un diagrama de tornado, que permite visualizar el impacto de cada variable en la estimación final de costos.

## Resultados

### Costos totales entre 1957 y 1969

El costo total de la implementación del programa clásico de erradicación de la malaria en Colombia fue de USD $41’788.924, valor que incluye aportes nacionales e internacionales (cuadros 1 y 2). El costo promedio anual del programa durante el período analizado fue de USD $3’214.533 (IC _95 %_: 3’020.280 a 3’408.785).

**Cuadro 1:** Costos de la Campaña de Erradicación de la Malaria en Colombia por fuente de financiación, 1957-1969

Año	Fuentes nacionales	Fuentes internacionales			Total
		USAID	UNICEF	OPS/OMS	
1957	616.981	-	-	160.000	776.981
1958	3’342.320	1’326.000	1’430.000	160.465	6’258.785
1959	3’226.017	-	600.000	165.000	3’991.017
1960	3’200.010	-	600.000	165.000	3’965.010
1961	3’187.685	-	600.000	169.000	3’956.685
1962	2’675.167	-	600.000	170.000	3’445.167
1963	1’296.638	-	641.913	158.000	2’096.551
1964	936.461	-	588.174	160.417	1’685.052
1965	702.346	-	541.913	159.417	1’403.676
1966	2’458.000	-	349.000	202.000	3’009.000
1967	2787.000	-	469.000	207.000	3’463.000
1968	3’108.000	-	369.000	156.000	3’633.000
1969	3’432.000	-	492.000	181.000	4’105.000
Total	30’968.625	1’326.000	7’281.000	2’213.299	41788.924

Los costos están expresados en dólares (USD).

**Cuadro 2.** Costos de las fases de la Campaña de Erradicación de la Malaria en Colombia, 1957-1969

Organización ejecutiva	Dirección de Campañas Directas	Servicio Nacional de Erradicación de la Malaria		Servicio Nacional de Salud Pública	Total
Fase	Preparatoria	Ataque	Consolidación	Mantenimiento	
Período	1957-1958	1959-1962	1963-1965	1966-1969	1957-1969
Costos (USD)	776.981	33’603.645	5’985.279	1’423.019	41788.924

**Figura 1.**
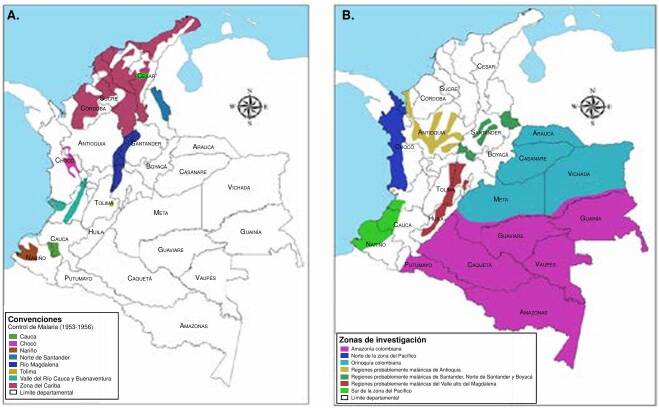
Evolución del control de la malaria en Colombia. A) Zonas de control de malaria previas a la Campaña de Erradicación de la Malaria (1953-1956); B) Áreas de investigación y control incluidas en la Campaña de Erradicación de la Malaria, además de las existentes en el programa de control

La figura 1 muestra una representación del Programa de Control de la Malaria, implementado entre 1953 y 1956, así como las áreas donde posteriormente se implementó la Campaña de Erradicación de la Malaria. Aunque se añadieron nuevas zonas de intervención, se continuaron las actividades en las áreas originalmente incluidas en el Programa de Control de la Malaria.

### Tendencia del gasto 1957-1969

**Figura 2.**
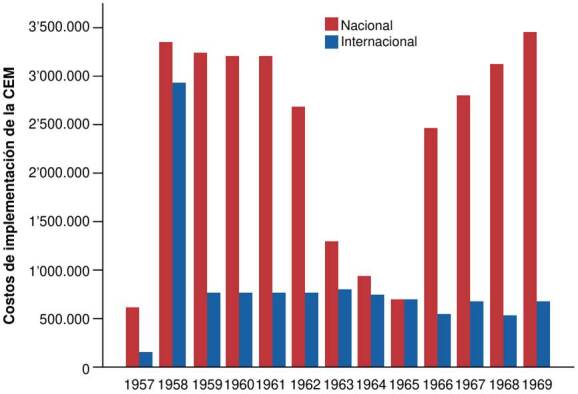
Comportamiento de los costos anuales de la implementación de la Campaña de Erradicación de la Malaria en Colombia, 1957-1969

El análisis del comportamiento anual de los gastos muestra que, hasta 1962, los aportes nacionales e internacionales mantuvieron una tendencia estable. Sin embargo, entre 1963 y 1965, se observó una reducción drástica en la inversión, seguida de una recuperación parcial de los recursos nacionales a partir de 1966. En contraste, los aportes internacionales disminuyeron significativamente en esta etapa (figura 2).

### Distribución de costos según fuentes de financiación

Del total de costos de implementación, el 74,1 % (USD $30’968.625 / USD $41788.924) provino de fuentes nacionales de financiación. La financiación internacional contribuyó principalmente a la adquisición y el suministro de insumos críticos, como medicamentos, exámenes diagnósticos, insecticidas, equipos, parque automotor y asistencia técnica. El mayor aporte internacional se concentró entre 1958 y 1962 y equivalió a USD $5’985.465. Esta cifra representó el 53,1 % del total de aportes internacionales recibidos en todo el período (cuadro 1).

**Figura 3.**
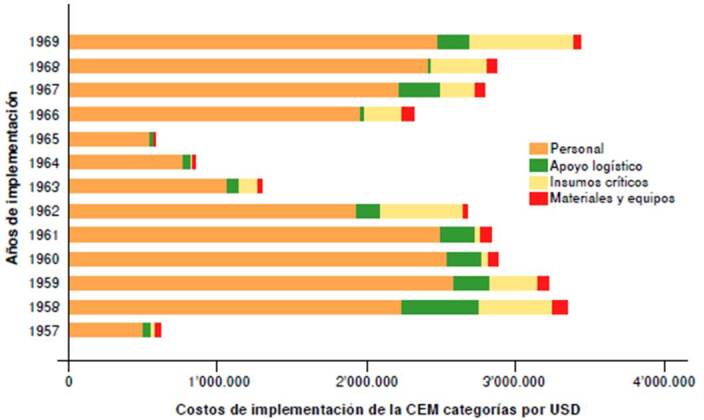
Distribución anual del presupuesto en la Campaña de Erradicación de la Malaria en Colombia, 1957-1969

El rubro más significativo fue el de personal, en el que se invirtió el 76,8 % (USD $23795.690 / USD $30’968.625) del presupuesto total proveniente de fuentes de financiación nacionales (cuadro 3, figura 3).

**Cuadro 3.** Estructura de costos de la Campaña de Erradicación de la Malaria en Colombia financiados con fondos nacionales, discriminado por ítem, 1957-1969

Año	Personal	Apoyo logístico	Insumos críticos	Materiales y equipos	Total
1957	506.541	46.274	25.296	38.870	616.981
1958	2’238.301	516.833	487.882	99.304	3’342.320
1959	2’587.662	242.895	318.452	77.008	3’226.017
1960	2’540.907	236.831	348.520	73.753	3.200.011
1961	2’497.967	231.025	385.150	73.542	3187.684
1962	1’937.956	161.365	546.457	29.389	2’675.167
1963	1’068.430	79.095	128.367	20.746	1’296.638
1964	768.834	64.616	92.710	10.301	936.461
1965	555.556	92.710	44.950	9.130	702.346
1966	1’966.400	159.770	255.632	76.198	2’458.000
1967	2218.452	275.913	234.108	58.527	2787.000
1968	2’427.348	233.100	379.176	68.376	3’108.000
1969	2’481.336	212.784	696.696	41.184	3’432.000
Total	23795.690	2’553.211	3’943.396	676.328	30’968.625

### Distribución de costos por fases durante las etapas de erradicación

La fase preparatoria, realizada en un año, tuvo un costo de USD $776.981, de los cuales el 79,4 % fue financiado con recursos nacionales. Las fases de ataque (1958-1962) y mantenimiento (1966-1969), representaron el 94,7 % del presupuesto del período, con costos de USD $33’603.645 y USD $5’985.279, respectivamente. El resto del presupuesto se destinó a vigilancia y control de focos detectados en la fase de mantenimiento (cuadro 2).

### Resultados de la fase preparatoria

En esta fase se logró la caracterización, delimitación y distribución del problema palúdico en el país. Los datos obtenidos sirvieron de insumo básico para la planeación de las operaciones de rociamiento intradomiciliario en las viviendas situadas en áreas maláricas incluidas en el Plan de Erradicación de la Malaria en Colombia.

Entre los resultados de mayor relevancia, se encontraron:

- Área malárica = 970.849 km^2^
- Altitud de transmisión: 0 a 1.500 msnm
- Población en riesgo: 7’694.543 habitantes
- Promedio de casos estimados = 76.596 notificados
- Especies: *Plasmodium falciparum* (54 %), *P. vivax* (44 %) y *P. malariae* (2 %)
- Muertes = 1.576
- Vectores principales: *Anopheles albimanus*, *An. nuneztovari, An. darlingi, An. punctimacula* y *An. neivai*.

En términos de viabilidad de erradicación, se estableció que:

- El 20 % del territorio era área de fácil erradicación. Alrededor del 50 % correspondía a áreas donde eliminar la enfermedad era posible, pero requería esfuerzos significativos y sostenidos, como mejoras en el acceso a servicios de salud, control de vectores y tratamiento; el 30 % eran áreas donde eliminar la enfermedad era muy complicado debido a condiciones ecológicas favorables para el vector, acceso limitado a servicios de salud o transmisión alta y persistente.
- Estimación de la carga económica de la malaria: USD $30 millones anuales.

### Resultados de la fase de ataque

**Figura 4.**
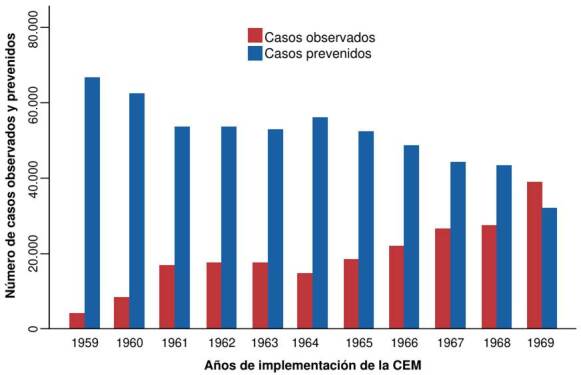
Casos observados y prevenidos en la Campaña de Erradicación de la Malaria en Colombia, 959-1969. Durante 1959-1962, se registraron 64.967 casos de malaria y se previnieron 290.188 casos, lo que equivale a 4,5 casos prevenidos por cada caso reportado.

Durante la fase de ataque inicial (octubre de 1958 a 1962), se alcanzaron coberturas de rociamiento intradomiciliario con DDT del 95 al 100 %. Se intervinieron 1 ’178.814 viviendas en diez ciclos semestrales. El costo total de esta fase fue de USD $22’393.645, con un costo unitario por vivienda de USD $1,89 (IC _95%_: 1,68 a 2,30) (cuadro 4). Durante este período, se registraron 64.967 casos de malaria y se previnieron 290.188 casos (figura 4), lo que resultó en una proporción de 4,5 casos prevenidos por cada caso registrado.

**Cuadro 4.** Costo anual del rociamiento intradomiciliario con dicloro-difenil-tricloroetano (DDT) durante la Campaña de Erradicación de la Malaria en Colombia, 1959-1969

Año	RID (n)	USD $	Valor unitario por casa
1959	2’357.627	3’991.017	1,69
1960	2’358.989	3’965.010	1,68
1961	2127.057	3’956.685	1,86
1962	1’431.774	3’445.167	2,41
1963	1’163.280	2’096.551	1,80
1964	871.294	1’685.052	1,93
1965	744.002	1’403.676	1,89
1966	677.228	3’009.000	4,44
1967	741.895	3’463.000	4,67
1968	916.892	3’633.000	3,96
1969	980.578	4105.000	4,19
Total	14’370.016	34’753.158	2,42*

RID: rociamiento intradomiciliario * Costo promedio por vivienda intervenida.”

Entre 1966 y 1969, se reanudó la operatividad de la fase de ataque tras haber sido suspendida entre 1963 y 1965. Se realizaron cuatro ciclos semestrales de rociamiento, con un costo total de USD $14’210.000. El costo promedio por vivienda intervenida fue de USD $4,3 (IC_95%_: 3,49 a 4,72). Durante este período, se registraron 114.967 casos de malaria y se previnieron 169.157 casos (cuadro 5, figura 4), lo que resultó en una proporción de 1,5 casos prevenidos por cada caso registrado.

**Cuadro 5.** Número de casos de malaria reportados y prevenidos durante la Campaña de Erradicación de la Malaria en Colombia, 1959-1969

Año	Casos reportados	Casos prevenidos	Relación casos prevenidos/ casos reportados
1959	4.172	66.859	16,02
1960	8.426	62.605	7,43
1961	16.974	54.057	3,18
1962	17.497	53.534	3,06
1963	17.898	53.133	2.97
1964*	14.728	56.303	3,82
1965*	18.240	52.791	2,89
1966**	22.148	48.883	2,21
1967**	26.570	44.461	1,67
1968**	27.286	43.745	1,60
1969**	38.963	32.068	0,82
Totales	212.902	568.439	2,67

* Durante 1964-1965 se notificaron 32.968 casos de malaria y se previnieron 109.094 casos, lo que equivale a 3,3 casos prevenidos por cada caso notificado.

** Entre 1966 y 1969, se registraron 114.967 casos de malaria y se previnieron 169.157 casos, lo que corresponde a 1,5 casos prevenidos por cada caso notificado.

### Resultados de la fase de mantenimiento y consolidación

El costo total de la vigilancia y el control de los focos residuales en la fase de mantenimiento fue de USD $5’985.279, equivalente al 14,3 % del presupuesto total. El costo promedio por vivienda intervenida fue de USD $2,70 (IC_95%_: 2,51 a 3,24). Durante esta fase, se observaron 32.968 casos de malaria y se previnieron 109.094 casos, lo que resultó en una proporción de 3,3 casos prevenidos por cada caso registrado (cuadro 5).

### Casos totales observados y prevenidos

Entre 1959 y 1969, se registraron 212.902 casos de malaria. Según la línea de base de incidencia esperada, se previnieron 568.439 casos en este período, lo que representó un ahorro estimado de USD $98’398.546 (cuadro 5).

### Resultados del análisis de sensibilidad

**Figura 5.**
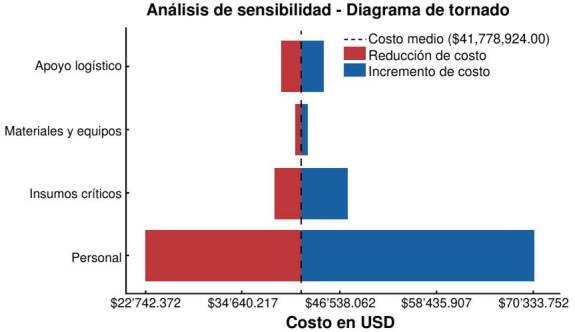
Análisis de sensibilidad univariado. Se muestran los parámetros que más influyen en el costo por caso prevenido de malaria durante la Campaña de Erradicación (1957-1969).

Se observó que la variable con mayor influencia en el análisis de sensibilidad fue el costo del personal, con un rango de variación entre USD $22742.372 y $70’333.752, lo que indica que los ajustes en este rubro generaron los cambios más significativos en el presupuesto global. una variación entre USD $38’624.207 y $47’496.848, lo que refleja la gran sensibilidad del costo total ante fluctuaciones en su disponibilidad y precio. Los materiales y equipos presentaron una variabilidad reducida en comparación con otras categorías, con un rango entre USD $41’170.228 y $42’590.517, lo que sugiere una menor sensibilidad a los cambios en este rubro dentro del modelo de costos. Finalmente, el apoyo logístico presentó el menor impacto en la estimación total, con un rango entre USD $39’481.034 y $44’587.456, lo que sugiere que las modificaciones en este componente no afectaron significativamente el presupuesto total del programa (figura 5).

## Discusión

La presente evaluación económica de la Campaña Nacional de Erradicación de la Malaria evidenció que, a pesar de la contribución financiera y el respaldo político del gobierno colombiano, no se pudo lograr su erradicación en el país. Aunque se registró una reducción importante e histórica en los niveles endémicos de la enfermedad en el período evaluado -lo que representó un ahorro considerable-, este logro no tuvo la sostenibilidad requerida. Situaciones similares se evidenciaron en la mayoría de los países endémicos y regiones con gran carga de la enfermedad a nivel mundial, como África, donde se adoptó la política global de erradicación de la malaria ^15^^,^^16^.

El valor social y económico de la Campaña de Erradicación de la Malaria era el argumento principal que justificaba su adopción en lugar de los programas de control. La idea de que los costos de la campaña representarían una inversión de capital por un tiempo limitado y no un gasto ordinario permanente, se puso en tela de juicio casi de inmediato; no solo por la comprensión progresiva de los costos elevados que generaba la vigilancia, sino porque se postuló sin tener en cuenta el principio de descontar el valor presente de los gastos futuros.

La consecuencia más grave del fracaso de la meta de erradicación fue el retiro del apoyo financiero de la UNICEF, la *U. S. Agency for International Development* (USAID) y otros organismos que respaldaban las campañas de erradicación en los diferentes países ^17^^,^^18^. Por esta razón, los gobiernos tuvieron que asumir el déficit mediante créditos externos de organismos internacionales y préstamos internos. Al mismo tiempo, el aumento del costo de los insumos críticos (insecticidas), la infraestructura tecnológica y la logística, redujo drásticamente la capacidad operativa de los programas, la calidad de las actividades y la moral del personal ^19^.

Durante los años 1954,1955 y 1956, la Organización Sanitaria Panamericana estableció que las asignaciones presupuéstales adoptadas por los países de las Américas para los programas de control antimalárico, fueron de USD $10’620.402, $11’808.110 y $14’889.439, respectivamente. En Colombia, las apropiaciones presupuéstales del gobierno para los mismos años fueron de USD $799.375, $665.750 y $402.769 ^19^. De la operatividad de las actividades se encargó la División de Malariología del Ministerio de Salud Pública. A su vez, para poder garantizar la implementación de las campañas de erradicación de la malaria, los gastos que asumieron los países fueron más del doble de los aportados por los organismos de cooperación internacional. Se estima que, en las fases iniciales de la campaña, los recursos económicos de apoyo internacional fueron de USD $764 millones y, para el periodo completo, se calculó en cerca de USD $1.500 millones ^20^^,^^21^.

La tendencia de los costos se mantuvo relativamente estable ya que, en el lapso inicial, se garantizó la disponibilidad y la apropiación oportuna de los recursos nacionales e internacionales necesarios para la implementación de la fase de ataque entre 1959 y 1963. Sin embargo, el descenso de los costos entre 1963 y 1965, se atribuye a la reducción de los recursos requeridos para la continuidad operativa de la campaña. Esta disminución se debió a la creciente evidencia y a las proyecciones que anticipaban que no se lograría la meta de interrupción de la transmisión, lo que condujo al retiro del apoyo financiero internacional.

Aunado a lo anterior, el peso colombiano se devaluó debido a la crisis económica mundial y la inflación monetaria de la época; hubo una colonización activa e intensa de regiones selváticas con elevado potencial de transmisión y se agudizaron los problemas de orden público que restringían o, incluso, impedían las actividades de control antimalárico en las áreas objeto de la campaña. Además, la topografía accidentada y el difícil acceso a áreas rurales dispersas -marcadas por procesos socioeconómicos y culturales complejos- afectaron aún más la operatividad y agravaron la situación epidemiológica ^21^.

El retiro gradual de los aportes de UNICEF contribuyó a una situación crítica en la disponibilidad de insumos clave, como insecticidas, vehículos, equipos y otros materiales, necesarios para cumplir con los planes operativos definidos. Las dificultades en la adquisición e importación de insecticidas y otros insumos esenciales, el incremento constante del costo de bienes y servicios, la renuencia marcada de la población frente a las operaciones de rociamiento intradomiciliario y la insuficiencia de personal operativo, explican la interrupción de la operación de la campaña en la tercera parte del área malárica y su disminución considerable en el resto del país ^22^^,^^23^.

La fase de ataque era el eje fundamental de la Campaña de Erradicación de la Malaria y requería una importante inversión del presupuesto del plan de operaciones para garantizar su cobertura total. Sin embargo, los insumos necesarios para llevarla a cabo -como insecticidas, equipos, vehículos, medicamentos y otros- resultaron ser los más costosos, ya que eran importados por UNICEF y USAID. Además, el recurso humano operativo requería inversiones oportunas en transporte, viáticos y otros ^24^.

Durante los años posteriores a la fase de ataque, los niveles endémicos que se habían reducido comenzaron a recuperarse en las áreas selváticas de colonización. Se registraron brotes epidémicos en las áreas en fase de consolidación y las actividades de vigilancia eran insuficientes por la escasa disponibilidad de recursos. Dicha situación se prolongó hasta 1966, cuando se obtuvieron nuevos recursos, se intensificó la fase de ataque y se restableció la vigilancia. Al año siguiente, se restableció la operación en casi toda el área interrumpida y se regresó a la fase de ataque en regiones que estaban en proceso de consolidación, pero que se habían reinfectado ^21^.

En los últimos años, la iniciativa de eliminación de la malaria ha sido renovada y posicionada políticamente (con pequeños cambios), pero la información económica continúa siendo la evidencia esencial más sólida y de mayor peso que respalda esta decisión ^25^^,^^26^. Colombia se adhirió a la iniciativa de eliminación de la malaria y la adoptó en su Plan Estratégico Nacional 2016-2030 ^27^.

Entre 1980 y 1990, se llevaron a cabo estudios para evaluar los costos económicos de la malaria en los países endémicos de las Américas. En Perú, la malaria representó un alto costo económico en 1998 -equivalente al 3 % del producto interno bruto- en las regiones de Loreto, Piura y Tumbes, donde la incidencia es mayor. El gasto estatal asignado al control de la malaria fue solo el 1 % del gasto público en salud ^28^. En Brasil, se observó una reducción del 92 % en los años de vida ajustados por discapacidad (AVAD) atribuibles a la malaria entre 1990 y 2017. La tasa de AVAD por 100.000 habitantes disminuyó de 42,5 (IC_95%_: 16,6 a 56,9) en 1990 a 3,4 (IC_95%_: 2,7 a 4,7) en el 2017 ^29^.

En Colombia, se realizó una estimación preliminar del costo de la Campaña de Erradicación de la Malaria entre 1957 y 1969, que se situó en aproximadamente USD $63’554.908. Sin embargo, estos resultados estaban sobredimensionados debido a sesgos en la conversión a dólares (derivados del uso de tasas oficiales en ciertos años) y a la falta de fiabilidad de algunas fuentes de información consultadas (Padilla JC, Acuña LM, Olivera M. ¿Cuánto costó la Campaña de Erradicación de la Malaria en Colombia, 1957-1979? Biomédica. 2022;42(Supl. 3):199-200. Memorias del XVIII Congreso Colombiano de Parasitología y Medicina Tropical. Bogotá, D. C., 30 de noviembre al 3 de diciembre de 2022). Un estudio más reciente reveló que el costo promedio de la atención de la malaria no complicada en 2019 fue de USD $17,4. Según la especie parasitaria, los costos de atención de los casos no complicados variaron entre USD $17,3 y $22,5. En contraste, el costo promedio de la atención de la malaria complicada fue de USD $593,8 ^30^.

El impacto económico de la malaria ha sido objeto de estudios en los cuales se ha encontrado una asociación significativa entre la reducción de su incidencia y el crecimiento económico. En 1995, se observó que los países con gran prevalencia de malaria tenían niveles de ingresos equivalentes solo al 33 % de los países no endémicos, independientemente de si se encontraban en África o no. Sin embargo, tras la eliminación de la malaria, estos países experimentaron un mayor crecimiento económico en comparación con sus vecinos. Por ejemplo, se encontró que una reducción del 10 % en la incidencia de la malaria se asociaba con un incremento del 0,3 % en el producto interno bruto ^25^.

Estos hallazgos resaltan la importancia de abordar la malaria desde una perspectiva económica, ya que el control de la enfermedad puede tener un impacto positivo en el desarrollo de los países afectados. Asimismo, en otro estudio se analizó el impacto potencial de reducir la incidencia de la malaria entre el 2016 y el 2030, y se propuso que aumentar la inversión para reducir su incidencia y mortalidad podría mejorar la productividad de la fuerza laboral, al recuperar los días de trabajo perdidos debido a la enfermedad y al cuidado de familiares enfermos ^31^.

Se puede concluir que la evaluación de costos y resultados de la Campaña de Erradicación de la Malaria en Colombia mostró que, a pesar del apoyo político, la cooperación técnica internacional, la disponibilidad y la oportunidad de los recursos requeridos, no se alcanzó la meta de interrupción de la transmisión de la malaria. Las explicaciones institucionales sobre el fracaso de esta campaña señalan tres factores clave. El primero, las limitaciones técnicas, entre ellas, la resistencia de *P. falciparum* a la cloroquina, la exofilia de los vectores y su resistencia al DDT en algunas regiones. El segundo, los problemas operacionales, como las dificultades de acceso a zonas endémicas, restricciones presupuestarias, gran rotación de personal y precariedad laboral, además de fallas logísticas y limitada disponibilidad de insumos, que afectaron la ejecución de las estrategias. El tercero, los factores ambientales y sociales, entre los que se destacan el desplazamiento poblacional, la deforestación y la falta de continuidad en las políticas de control, factores determinantes que contribuyeron a la persistencia de la enfermedad. Durante la ejecución de la campaña, se observaron resultados interesantes y prometedores en la reducción de la endemia, aunque no se logró garantizar su sostenibilidad en el tiempo ^17^^,^^32^.

Algunas limitaciones del presente estudio incluyen las dificultades para obtener información confiable y consolidada de las fuentes oficiales nacionales e internacionales. En contraste, el aporte de este estudio es que permite extraer lecciones clave para optimizar las estrategias actuales de eliminación de la malaria en el país, resaltándose la necesidad de modelos integrales, sostenibles y costo-efectivos en futuras intervenciones.

Se recomienda desarrollar investigaciones sobre el tema con el fin de profundizar y comprender mejor la etapa de erradicación de la malaria en Colombia, e impulsar la realización de estudios de evaluación económica (eficacia, efectividad y equidad) para generar evidencias nacionales que apoyen la toma de decisiones. Se deben contemplar las lecciones aprendidas durante la implementación de la Campaña de Erradicación de la Malaria (y otros proyectos), para aplicarlas en los planes actuales de eliminación de la enfermedad. Por ejemplo, se debe focalizar y estratificar el riesgo de malaria en las diferentes regiones del territorio nacional, mejorar la capacidad de gestión técnica y operativa del Programa de Enfermedades Transmitidas por Vectores en los diferentes niveles e implementar un modelo integral, liderado por el sector salud y respaldado por el compromiso de todos los actores, que priorice la prevención y la promoción, y garantice la regularidad y sostenibilidad de las acciones.

## References

[1] Word Health Organization (2024). World malaria report 2024.

[2] Olivera MJ, Padilla JC, Chaparro PE, León W (2024). Epidemiology of Plasmodium vivax malaria infection in Colombia. Microbe.

[3] Ferro C, Rincón C, Olivera MJ, Walteros DM, Prieto F (2025). Clinical and epidemiological characteristics of complicated malaria in Colombia, 2019. The Microbe.

[4] Quevedo E, Borda C, Eslava JC, Garcia CM, Guzmán MDP, Mejia P (2004). Café y gusanos, mosquitos y petróleo: el tránsito desde la higiene hacia la medicina tropical y la salud pública en Colombia, 1873-1953.

[5] Padilla JC, Olivera MJ, Padilla MC (2022). Epidemiological evolution and historical anti-malarial control initiatives in Colombia, 1848-2019. Infez Med.

[6] Zozaya C. (1941). Informe sobre organización de la lucha antipalúdica en Colombia. Rev Hig.

[7] Russell PF. (1953). Paludismo. Compendio de principios básicos.

[8] Ministerio de Salud Pública (1956). Plan de erradicación de la malaria en Colombia.

[9] Organización Mundial de la Salud (1958). Los diez primeros años de la Organización Mundial de la Salud.

[10] Soper FL. (1955). La erradicación de la malaria en el hemisferio occidental. Bol Sanit Panam.

[11] Organización Panamericana de la Salud (1963). La malaria en las Américas: bosquejo de la batalla que libra el hemisferio para terminar con un viejo enemigo.

[12] Ministerio de Salud (1982). Dirección de Campañas Directas. El programa de Malaria en Colombia, 1979-1982.

[13] Comisión Económica para América Latina y el Caribe (CEPAL) (1994). Serie histórica del crecimiento económico de América Latina, 1950-1993.

[14] Departamento Administrativo Nacional de Estadística (DANE) (1960). Boletín Mensual de Estadística.

[15] Owens PN. (1958). El programa mundial de erradicación de la malaria. Bol Sanit Panam.

[16] Mills A, Lubell Y, Hanson K (2008). Malaria eradication: The economic, financial, and institutional challenge. Malar J..

[17] Padilla JC, Olivera MJ, Chaparro P, Quiñónez ML, Escobar JP, Álvarez G (2022). La campaña de erradicación de la malaria en Colombia, 1959-1979. Biomédica.

[18] Nájera J. (1991). El paludismo y las actividades de la Organización Mundial de la Salud. Bol Sanit Panam.

[19] Organización Mundial de la Salud (1957). Comité de expertos en paludismo: sexto informe.

[20] Organización Mundial de la Salud (2021). Erradicación del paludismo: beneficios, perspectivas futuras y viabilidad. Informe del grupo consultivo estratégico sobre erradicación del paludismo.

[21] Ministerio de Salud (1982). Dirección de Campañas Directas. El programa de Malaria en Colombia, 1979-1982.

[22] Alvarado CA, Davée RL (1955). Informes sobre los programas de erradicación de la malaria en las Américas.

[23] Bruce-Chwatt LJ. (1978). El costo de la malaria y su control en relación con la realidad socioeconómica. Bol Sanit Panam.

[24] Servicio Nacional de Erradicación de la Malaria (1968). Informe del director del SEM a la Vil Reunión del Servicio Nacional de Erradicación de la Malaria de América del Sur.

[25] Gallup JL, Sachs JD (2001). The economic burden of malaria. Am J Trop Med Hyg.

[26] Sarma N, Patouillard E, Cibulskis RE, Arcand JL (2019). The economic burden of malaria: Revisiting the evidence. Am J Trop Med Hyg.

[27] Ministerio de Salud y Protección Social (2021). Plan Estratégico Nacional para el Control y la Eliminación de la Malaria 2016-2030.

[28] Francke P. (2003). Impacto económico de la malaria en el Perú. Economía.

[29] Bezerra JMT, Barbosa DS, Martins-Melo FR, Werneck GL, Braga EM, Tauil PL (2020). Changes in malaria patterns in Brazil over 28 years (1990-2017): Results from the global Burden of Disease Study 2017. Popul Health Metr.

[30] Olivera MJ, Padilla JC, Cárdenas IM (2023). A propensity score matching analysis using statistical methods for estimating the impact of intervention: The cost of malaria and its impact on the health system. Health Anal.

[31] Patouillard E, Han S, Lauer J, Barschkett M, Arcand JL (2023). The macroeconomic impact of increasing investments in malaria control in 26 high malaria burden countries: An application of the updated EPIC model. Int J Health Policy Manag.

[32] Padilla JC, Olivera MJ, Acuña L, Chaparro P (2024). Changes in the endemic-epidemic pattern of malaria in Colombia, 1978-2021. Rev Soc Bras Med Trop.

